# Current status of immunological therapies for rheumatoid arthritis with a focus on antigen-specific therapeutic vaccines

**DOI:** 10.3389/fimmu.2024.1334281

**Published:** 2024-03-06

**Authors:** Daniel H. Zimmerman, Zoltan Szekanecz, Adrienn Markovics, Kenneth S. Rosenthal, Roy E. Carambula, Katalin Mikecz

**Affiliations:** ^1^ CEL-SCI Corporation, Vienna, VA, United States; ^2^ Department of Rheumatology, Faculty of Medicine, University of Debrecen, Debrecen, Hungary; ^3^ Department of Orthopedic Surgery and Department of Internal Medicine, Division of Rheumatology, Rush University Medical Center, Chicago, IL, United States; ^4^ Department of Basic Sciences, Augusta University/University of Georgia Medical Partnership, Athens, GA, United States; ^5^ Department of Orthopedic Surgery, Rush University Medical Center, Chicago, IL, United States

**Keywords:** peptide vaccine, immunotherapy, rheumatoid arthritis, cytokines, proteoglycan (PG, aggrecan), PG-induced arthritis, collagen-induced arthritis

## Abstract

Rheumatoid arthritis (RA) is recognized as an autoimmune joint disease driven by T cell responses to self (or modified self or microbial mimic) antigens that trigger and aggravate the inflammatory condition. Newer treatments of RA employ monoclonal antibodies or recombinant receptors against cytokines or immune cell receptors as well as small-molecule Janus kinase (JAK) inhibitors to systemically ablate the cytokine or cellular responses that fuel inflammation. Unlike these treatments, a therapeutic vaccine, such as CEL-4000, helps balance adaptive immune homeostasis by promoting antigen-specific regulatory rather than inflammatory responses, and hence modulates the immunopathological course of RA. In this review, we discuss the current and proposed therapeutic products for RA, with an emphasis on antigen-specific therapeutic vaccine approaches to the treatment of the disease. As an example, we describe published results of the beneficial effects of CEL-4000 vaccine on animal models of RA. We also make a recommendation for the design of appropriate clinical studies for these newest therapeutic approaches, using the CEL-4000 vaccine as an example. Unlike vaccines that create or boost a new immune response, the clinical success of an immunomodulatory therapeutic vaccine for RA lies in its ability to redirect autoreactive pro-inflammatory memory T cells towards rebalancing the “runaway” immune/inflammatory responses that characterize the disease. Human trials of such a therapy will require alternative approaches in clinical trial design and implementation for determining safety, toxicity, and efficacy. These approaches include adaptive design (such as the Bayesian optimal design (BOIN), currently employed in oncological clinical studies), and the use of disease-related biomarkers as indicators of treatment success.

## 1 Introduction

**Figure 1:**
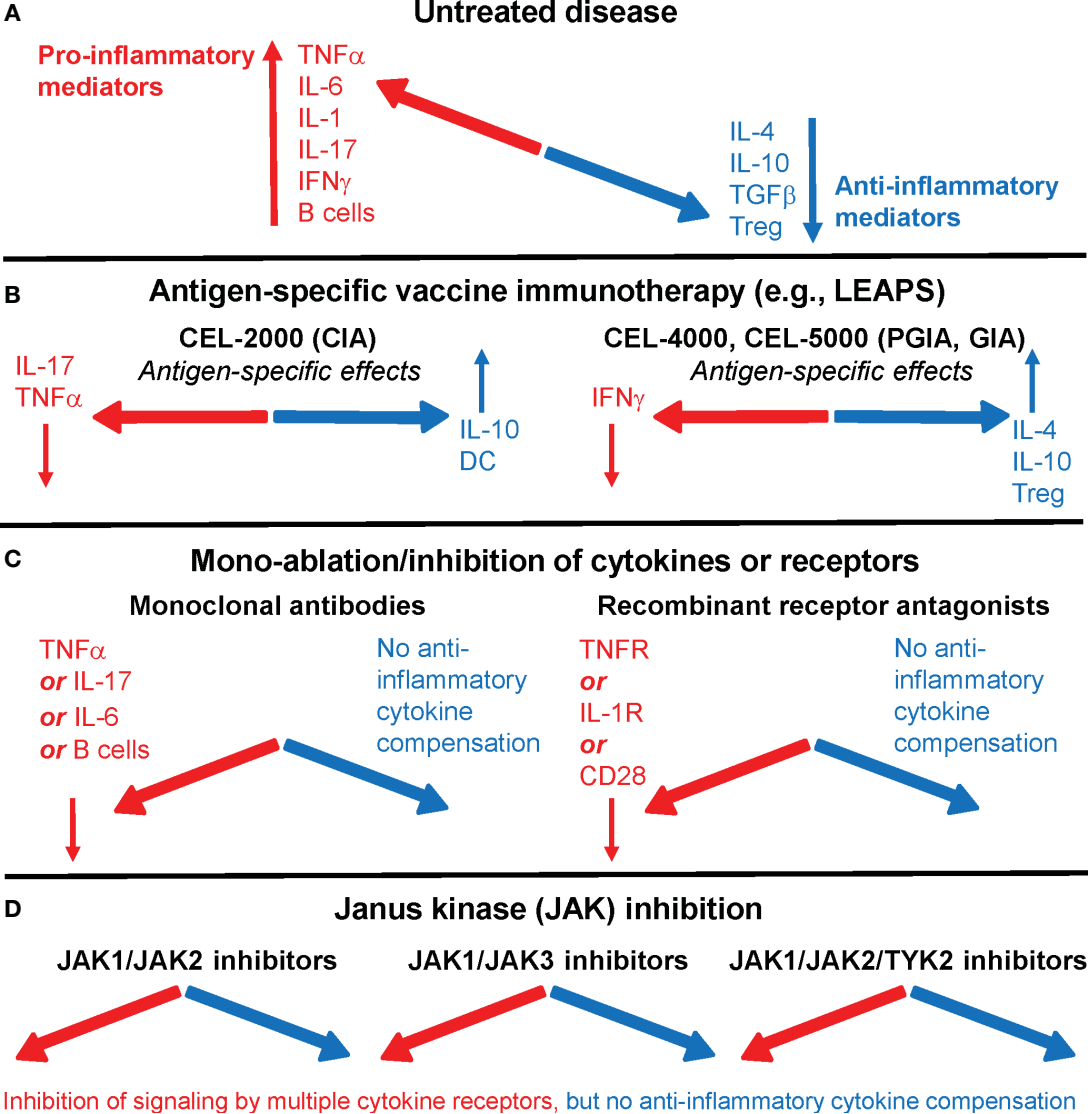
Immune system imbalance in rheumatoid arthritis (RA) or animal models of the disease and the effects of therapies on pro-inflammatory or anti-inflammatory mediators. **(A)** In untreated RA, pro-inflammatory mediators (red) overwhelm anti-inflammatory/regulatory mediators (blue). **(B)** Antigen-specific vaccine immunotherapy, as represented by Ligand Epitope Antigen Presentation System (LEAPS) immunotherapy in animal models of RA, restores immune balance. CEL-2000 LEAPS vaccine treatment of the collagen-induced arthritis (CIA) model of RA (left-side panel); CEL-4000 and CEL-5000 LEAPS vaccine treatments of the cartilage proteoglycan-induced and proteoglycan G1 domain-induced arthritis (PGIA and GIA) models (right-side panel) act in an antigen-specific fashion in these animal models of RA. **(C)** Current RA therapeutics using mono-ablation or inhibition of pro-inflammatory mediators, while reducing disease severity in most patients, introduce a different kind of immune imbalance. Monoclonal antibody treatment (left-side panel) directed against TNFα (e.g., infliximab), IL-6 receptor (tocilizumab), or B cells (rituximab), and recombinant receptor antagonists (right-side panel) induce systemic immune suppression without increasing the output of anti-inflammatory mediators. **(D)** Oral treatments with Janus kinase (JAK) inhibitors prevent intracellular pro-inflammatory cytokine signaling by using different classes of JAK inhibitors (e.g., tofacitinib, filgotinib, or baricitinib), but do not correct the deficiency of anti-inflammatory mediator production in RA. For more details, see references (2–5).

Currently used targeted or biological therapies in rheumatoid arthritis (RA) are effective and safe in most patients but increase the risk to certain infections. There is an unmet need for novel antigen-specific therapeutic approaches. In an earlier article, we looked at therapeutic vaccines for RA (1), and later we compared advanced targeted approaches (2) that are currently being used or proposed for the treatment of RA, including our Ligand Epitope Antigen Presentation System (LEAPS) technology (**Figure 1**) (6, 7).

Many of the current approaches use monoclonal antibodies (mAbs) or soluble cytokine receptor antagonists to ablate a single cytokine, such as tumor necrosis factor (TNF)α, interleukin (IL)-1, or IL-6, or interfere with a specific T helper cell response (Th1 or Th17) by targeting the cytokines that initiate or are being produced during autoimmune/inflammatory responses in RA. Another ablative approach targets a cell surface marker (CD20) of B cells, to reduce pathological antibody production and stop the cycle of B cell-driven antigen-specific T cell activation, via elimination of B cells by an anti-CD20 mAb. These treatments are referred to as biological Disease Modifying Anti-Rheumatic Drugs (DMARDs).

More recent therapies target intracellular second messenger pathways that are activated by inflammatory cytokines by inhibiting one or more Janus kinase (JAK) family protein tyrosine kinases. The JAKs phosphorylate signal transducer and activator of transcription (STAT) proteins, which then dimerize, translocate to the nucleus, and activate genes coding for cytokines and other inflammatory mediators. The JAK inhibitors are orally administered small molecules (3).

To gain deeper insight into the rationale of current therapeutic approaches, the reader is directed to recent reviews on RA in general (8), the role of biomarkers in the disease (9), and preventative strategies in at-risk individuals for RA (10), RA vaccines (11), and RA animal models (12). For currently approved RA therapeutics and recommendations for their clinical use, see published guidelines by the European Alliance of Associations for Rheumatology (EULAR, formerly the European League Against Rheumatism) and the American College of Rheumatology (ACR) for the management of RA using synthetic and biological DMARDs (13, 14). We also direct the reader to publicly available online sources but urge caution as some sites may include “off-label” use of drugs intended for RA patients.

Immunologically, RA is driven by antigens, multiple cytokines (such as TNFα, IL-6, IL-17, IL-23, IFNγ, IL-1), and a variety of cells including T cells, B cells, macrophages, neutrophils, osteoclasts, and synovial fibroblasts. Current United States Food and Drug Administration (FDA)-approved treatments mainly focus on one component of this multicomponent disease. All of these existing therapeutic approaches are systemic and as such, they compromise immune responses that are important for preventing certain new infections or the recurrence of chronic infections, especially tuberculosis, herpes viruses, and hepatitis B and C (15). In addition, treatment with certain JAK inhibitors might be associated with an increased risk of malignancy compared to anti-TNFα therapy (16), and can also increase the risk of venous thromboembolic disease (17).

Numerous autoantigens have been implicated as potential targets for antigen-specific therapeutic interventions in RA. As the immune-mediated damage of joint cartilage is a universal feature of RA, initial studies have identified articular cartilage molecules such as type II collagen (CII), the large aggregating proteoglycan (PG, aggrecan), and chondrocyte glycoprotein 39 as autoantigens [reviewed in (18, 19)]. The autoantigen repertoire was extended to proteins present not only in the joints, but also at extraarticular locations, including autologous serum IgG-antigen immune complexes, heat shock proteins, glucose-6-phosphate isomerase (GPI) and some others (18, 19). It is important to note that arthritis can be induced in experimental animals by immunization with cartilage CII or PG [reviewed in (12)] but not by immunization with other putative RA autoantigens. However, passive transfer of GPI-reactive serum from arthritic KBxN mice to non-diseased naïve animals can induce arthritis in the recipients (12). Nonetheless, the relationship between adaptive immune reactivity to these autoantigens and joint pathology in RA (or animal models) has not been completely understood (18). A paradigm shift occurred in the field of autoimmunity associated with RA with the discovery of anti-citrullinated protein antibodies (ACPAs) in the serum of a large proportion of RA patients (20). While serum ACPA helped develop an important diagnostic tool for RA, current immunological views (21) suggest that the majority of citrullinated proteins represent neoantigens (instead of autoantigens) that have undergone extensive post-translational modification (citrullination) and then triggered the production of widely cross-reactive ACPAs, and likely T cell responses, in RA patients. Thus far, attempts to identify Th cells that promote the production of singular antigen-specific ACPAs by B cells of RA patients have been met with limited success (21).

## 2 Antigen-specific and immunomodulatory therapies in RA and animal models

As an alternative to ablative therapies, immunomodulatory therapy represents an approach to rebalancing the immune system. Modulation of the functions of the adaptive immune system as a treatment of RA has been attempted using cell-based therapies or autoantigen-loaded liposomes (**Table 1**).

**Table 1:** Antigen-specific and immunomodulatory therapeutic approaches in RA.

Approach/ product	Rheumavax	DEN-181	MSC transplantation	Treg cell transfer	CAR-T or CAAR-T cell approach	CEL-4000 CEL-5000 vaccines
**Components**	Autologous DCs loaded *in vitro* with NF-κB inhibitor and liposome-encapsulated antigenic peptides	Liposomes encapsulating CII and calcitriol	Human umbilical cord blood MSCs	Peripheral blood Treg cells	T cells expressing chimeric citrullinated antigen or antibody receptors	LEAPS peptides (DerG-PG70 and DerG-PG275Cit) with adjuvant
**Immuno-** **modulatory action**	Increased Treg/Teff cell ratio	Tolerogenic effects on CII-specific and “bystander” Cit-Vim-specific T cells	Increased Treg/Th17 cell ratio	Suppression of Teff cell function	Targeting autoreactive ACPA producing B cells from RA patients	Increased Treg/Th1 ratio and correction of immune balance in favor of anti-inflammatory pathways
**Delivery**	ID or SC	SC	IV	IV	*In vitro*	SC
**Antigen specificity**	Citrullinated epitope peptides of CII, fibrinogen α and β chains, and vimentin	CII autoantigenic epitope peptide	–	Heat shock protein 70	Citrullinated CII, vimentin, fibrinogen, tenascin-C, and cyclocitrulline peptide-1	Proteoglycan antigenic epitope peptides, CEL-4000: PG70 CEL-5000: PG275Cit
**Preclinical studies published**	(22)	(23)	(24)	(25)	(26)	(6, 7)
Animal model	Antigen-induced arthritis (AIA)	PGIA	CIA	PGIA	–	PGIA, GIA
Outcome	Reduced arthritis severity, IL-10 and TGFβ-dependent suppression of pro-inflammatory cytokine production	Reduced disease severity, suppression of Teff cell function, induction of peripheral Treg cells	Reduced disease severity, suppression of M1 macrophage activation and promotion of M2 polarization	Reduced disease severity and suppression of Teff cells	Elimination (*in vitro* lysis) of RA B cells producing epitope- specific ACPA	Reduced disease severity and increased ratios of anti-inflammatory to pro-inflammatory cytokines
**Clinical trials**	Open-label Phase I (27)	Randomized Phase I (28)	Phase I (29)	–	–	–
Outcome	Reduced DAS28, and increased Treg/Teff cell ratios	Reduced disease activity	Reduced disease activity and pro-inflammatory cytokine levels	–	–	–
**Limitations**	Difficulties associated with the production of the DC-based delivery system	Probability of short-lived therapeutic effect	Short-lived effect, and potential for pathogenic conversion of MSCs	Rarity of Tregs, and potential for pathogenic Th1 or Th17 conversion	Potential CRS induction, and difficulties with the production of CAR-T cells	Uncertain percentage of RA patients recognizing autoantigenic PG peptides

DC, dendritic cell; Treg, regulatory T cell; Teff, effector T cell; ID, intradermal; SC, subcutaneous; Cit, citrullinated; PGIA, proteoglycan-induced arthritis; CII, type II collagen; Cit-Vim, citrullinated vimentin; MSC, mesenchymal stem cell; Th1, Th17, T helper 1 and, T helper 17 cells; IV, intravenous; CIA, collagen-induced arthritis; CAR, chimeric antigen receptor; CRS, cytokine release syndrome; GIA, proteoglycan G1 domain-induced arthritis.

Several preclinical and clinical trials have investigated the potential benefit of mesenchymal stem cell (MSC) transplantation in RA. For example, human MSCs have been reported to significantly attenuate collagen antibody-induced arthritis (CAIA) in mice (30). In clinical studies with RA patients, MSC therapy resulted in an increased T regulatory (Treg)/Th17 cell ratio (31, 32) and improved immune balance as indicated by decreased IL-1β, IL-6, IL-8 and TNFα, as well as increased production of IL-10 and transforming growth factor (TGF)β (29, 33). However, the potential of MSCs to differentiate into pathogenic cells *in vivo* is a serious concern in these studies (**Table 1**).

Based on the immunomodulatory effects of Treg cells, adoptive Treg cell transfer has gained attention in recent years. This approach requires the isolation, activation, and expansion of autologous or allogeneic Tregs prior to infusion back into the patient (34). In the collagen-induced arthritis (CIA) mouse model, adoptively transferred Tregs decreased disease severity and progression (35). Despite promising results, the clinical application of adoptive Treg transfer is hindered by the low occurrence of Tregs in peripheral blood, the probability of conversion into pathogenic T cells, and difficulties in processing the cells for therapy (36, 37) (**Table 1**). As an alternative, the safety and efficacy of a mAb that selectively activates Tregs has been investigated in a phase IIb, randomized, placebo-controlled clinical trial in patients with active RA, but did not demonstrate efficacy (38).

The clinical success of chimeric antigen receptor (CAR)-T cells in haemato-oncology boosted interest towards their application in autoimmune diseases. Recently, patients with refractory systemic lupus erythematosus (SLE) were treated successfully with CD19-directed CAR-T cell therapy to ablate B cells (39). In this promising study, CD19-targeting CAR-T cell treatment led to drug-free remission in SLE patients even after B cell recovery occurred post-treatment. One specific CAR-T cell approach proposed for RA is represented by the chimeric autoantibody receptor-T (CAAR-T) cells. These cells express an extracellular autoantigen recognized by the B cell receptor. The T cell-expressing autoantigen promotes binding to autoreactive B cells, subsequent CAAR-T cell activation and then lysis of the pathogenic B cells (40). It has been hypothesized that development of CAAR-T cells, expressing citrullinated antigens, would allow selective deletion of B cells producing anti-citrullinated protein Abs (ACPA, a hallmark of RA), while saving other B cells (41). In an RA study, four citrullinated peptide epitopes were selected as ligands for targeting autoreactive ACPA-producing B cells. Engineered T cells expressing a fixed anti-fluorescein isothiocyanate (FITC) CAR were constructed and tested for their ability to eliminate ACPA-specific autoreactive B cells. The anti-FITC CAR-T cells demonstrated the potential to recognize the corresponding FITC-labeled citrullinated peptide epitope and lyse autoreactive B cell subsets from RA patients *in vitro* (26). Nevertheless, the potential application of CAR- or CAAR-T cells in autoimmune diseases is limited by the various types of autoreactive responses driving RA, the different immune cells involved in RA, and serious side effects such as Cytokine Release Syndrome (CRS, also known as “cytokine storm”) as well as by the difficulty in preparing CAR- or CAAR-T cells (42) (**Table 1**).

Dendritic cell (DC)-based immunomodulatory approaches showed initial success in both animal models (22) and clinical trials in ACPA positive RA patients genetically predisposed to disease by possessing human leukocyte antigen-shared epitope (HLA-SE) risk alleles (27). Rheumavax, an intradermally administered vaccine was created from autologous DCs that were loaded with an irreversible NF-κB inhibitor (BAY11-7082) and exposed to liposomes containing four citrullinated peptide antigens (collagen type II_1237-1249_-Cit1240, fibrinogen α chain_717-725_-Cit720, fibrinogen β chain_433-441_-Cit436, and vimentin_447-455_-Cit450), respectively. Rheumavax (27) was administered once intradermally at two dose levels to HLA-SE and ACPA positive RA patients. This open-label phase I trial demonstrated a good safety and biological activity profile for Rheumavax as indicated by an increased ratio of Treg to T effector (Teff) cells and decreases in disease activity score in 28 joints (DAS28) within 1 month in Rheumavax-treated patients (**Table 1**). Again, although autologous cell therapies have been commercially available for almost a decade (43), to date, they have not been proven to be economically successful due to the extremely high cost of generating these patient-specific cells.

Another DC-targeting approach was tested in a double-blind, placebo-controlled exploratory phase I trial (28). DEN-181 vaccine, comprised of liposomes encapsulating a self-peptide collagen II_259-273_ (an autoantigen) and a NF-κB inhibitor 1,25-dihydroxycholecalciferol (calcitriol), was tested in RA patients in this trial. A single ascending dose of DEN-181 was administered subcutaneously to ACPA positive, HLA-SE positive RA patients treated with methotrexate only. DEN-181 was well tolerated and showed dose-associated immunomodulatory effects (28). In a pre-clinical animal study, the same group used a cartilage proteoglycan (PG, aggrecan)-derived peptide aggrecan_89-103_ as an autoantigenic epitope encapsulated into liposomes with or without calcitriol, and the liposomes were tested in the PG-induced arthritis (PGIA) model of RA. The authors reported that disease severity significantly decreased in an antigen-specific manner in mice receiving aggrecan_89-103_/calcitriol-containing liposomes every four days after disease onset (23) (**Table 1**).

**Figure 2:**
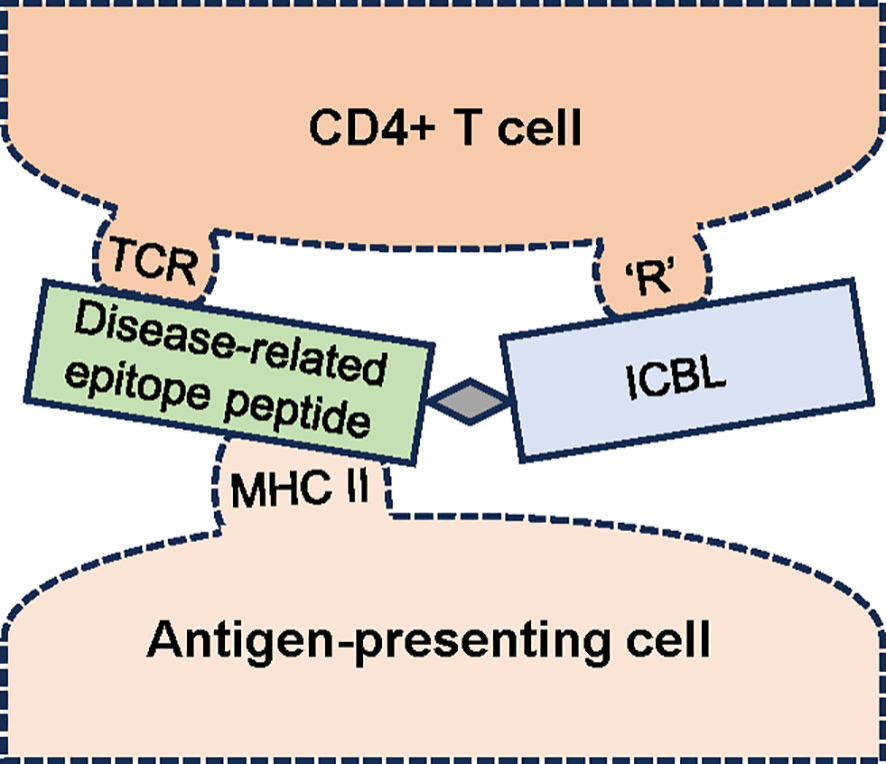
Schematic diagram of the interaction of an antigen-specific ligand epitope antigen presentation system (LEAPS) peptide conjugate with cognate CD4^+^ T cells. The LEAPS peptide is composed of an immune cell binding ligand (ICBL, blue rectangle), in this case, a DerG peptide derived from the β chain of human major histocompatibility complex (MHC) II. The ICBL moiety can bind to CD4 or another receptor (‘R’) on the T cell surface. DerG binding to CD4 modulates cellular activity. The disease-related epitope peptide (green rectangle), in this case, PG70 derived from human cartilage proteoglycan, is connected to the ICBL via a 3 amino acid spacer (grey diamond). The PG70 peptide is presented in the context of MHC II on the surface of the antigen-presenting cell (APC) to the T cell receptor (TCR). The binding of DerG-PG70 LEAPS peptide conjugate to cognate CD4^+^ T cells and APCs has been demonstrated in our earlier study (7).

As an easier-to-deliver mechanism for treating RA, we reported promising results with a peptide conjugate-based, antigen-specific immunomodulatory approach utilizing the LEAPS technology-based CEL-4000 vaccine (see **Figure 1**; **Table 1**). This approach promotes the rebalancing of pathogenic immune responses by inducing immunomodulation, not just by inducing tolerance or immune suppression, (44–47). CEL-4000 modulates the production of pro-inflammatory and anti-inflammatory/regulatory cytokines in a downward and an upward direction, respectively, and most importantly, in an antigen-specific manner. CEL-4000 incorporates the immunogenic PG70 epitope (from cartilage PG aggrecan) into a conjugate with the DerG LEAPS immune cell binding ligand (ICBL). The DerG ICBL peptide within the conjugate binds to, and acts on, CD4^+^ T cells. As illustrated in **Figure 2**, the disease (RA)-related epitope peptide moiety (PG70) of the LEAPS conjugate is presented to cognate CD4^+^ T cells through the engagement of MHC II on the surface of antigen-presenting cells (APC) and the TCR of the T cell while the DerG ICBL modulates the T cell activity through CD4 (48). Using fluorescence-labeled tetramers of the DerG-PG70 (CEL-4000) peptide for flow cytometry, our earlier study (7) demonstrated preferential binding of this conjugate to CD4^+^ T cells isolated from a PG-induced murine arthritis model of RA.

Thus, by engaging CD4^+^ T cells, CEL-4000 induces reduction or conversion of Th1 and Th17 cytokine production, resulting in a dominance of Th2 and Treg responses (6, 7). CEL-4000 was tested in the PGIA mouse model of RA (developed by Tibor T Glant and his colleagues) (49, 50). The PGIA and recombinant proteoglycan G1 domain-induced arthritis (GIA) models were used for the LEAPS studies for several reasons: (i) The diseased mice best resemble the pathology of RA; (ii) Like seropositive RA patients, the mice with PGIA or GIA produce ACPA and rheumatoid factor (RF); (iii) Arthritis development is more frequent in females than males; and (iv) Disease incidence increases with age. These features are not readily observed in other RA models, such as CIA or adjuvant/pristane-induced arthritis (AIA/PIA). Mice in our preclinical studies received three PG or recombinant human PG G1 domain (rhG1) antigen injections to initiate an early RA-like disease (PGIA or GIA) before treatment with the CEL-4000 vaccine. The first vaccine was administered subcutaneously right after the initial development of arthritis symptoms was noted in the fore- and hindlimbs, and a second dose was delivered in a similar way two weeks later. Improvement in disease severity, as assessed by visual arthritis scores (VAS), was noted at three weeks after the initiation of vaccine treatment, and the mice receiving CEL-4000 ended up with lower VAS as well as milder histological signs of joint inflammation than the controls receiving adjuvant only (7). CEL-4000 also modulated the animals’ immune responses by upregulating the production of anti-inflammatory/regulatory cytokines and downregulating the secretion of pro-inflammatory cytokines (**Table 1**). This was evident by detecting increases in IL-4, IL-10, and TGFβ levels and Foxp3^+^ Treg cells as well as decreased production of TNFα, IL-17 and IFNγ (6, 7). Presumably, such a rebalancing of the immune response cannot be observed in non-diseased (non-immunized, naïve) animals because we hypothesize that the CEL-4000 vaccine acts only on established, disease-driving and antigen-specific T cells (6, 7), which are not present or not active in naïve non-diseased animals or their healthy human counterparts. The antigen-specificity and immunomodulatory mechanism of the LEAPS vaccine has an advantage over the systemic ablative or inhibitory effects of DMARDs (mAbs or receptor antagonists, or the JAK inhibitors), as these currently used approaches compromise overall immunity and none of these therapies amend the dysregulation of the immune system that is observed in RA.

Additional developments have occurred for other forms of vaccines, including DNA vaccines, exosomes, Poly-lactic-co-glycolic acid (PLGP) particles, and epitope-modified (i.e., altered peptide ligand, APL) peptides, which have been examined in animal models of RA or other autoimmune diseases. A mRNA vaccine was tested in the autoimmune mouse model of multiple sclerosis (51) but similar tests have not yet been reported in animal models of RA. This is likely to change, based on the successes of mRNA vaccines against COVID-19. However, a mRNA vaccine requires a target antigen(s), but only a limited number of new RA-specific antigenic epitopes have been investigated, and the protective cytokines and immune responses in disease models of human RA (23, 27, 28, 44–47, 52–60) have not been recently revisited.

## 3 Preclinical studies of the CEL-4000 vaccine in mouse models of RA

For a vaccine therapy for RA to be effective, it must be able to act on the established pro-inflammatory and disease-driving immune responses, primarily on T cell-mediated immunity. As mentioned earlier, CEL-4000 vaccination successfully curtailed disease progression in mice already showing arthritis symptoms and immune reactions to PG (PGIA model) or to the recombinant G1 domain of PG (GIA model) (6, 7). The imbalance of immune responses in the diseased mice was demonstrated by increased (>1) ratios of pro-inflammatory cytokines such as IFNγ, IL-17, and TNFα, to anti-inflammatory/regulatory cytokines such as IL-4, IL-10, and TGFβ. This cytokine imbalance in the PGIA and GIA models of RA favored the pro-inflammatory Th1 and Th17 T cell responses over the Th2 and Treg regulatory responses. Disease progression was curtailed and the ratios were flipped in favor of the Th2 and Treg cytokines in mice treated with CEL-4000 (DerG-PG70, a conjugate of the DerG ICBL and the PG70 epitope peptides) but disease continued to progress in those treated with a LEAPS vaccine composed of a J ICBL and PG70 (J-PG70) (6, 7). Our recent studies showed that CEL-4000 (DerG-PG70), directed to the appropriate immune cells by the DerG ICBL, modulated the ongoing inflammatory immune response in an antigen (PG70)-specific manner, thus correcting the dysregulated immunological state involved in the disease. Since CEL-4000 is an antigen-specific vaccine, other immune responses should remain intact due to the absence of systemic immune suppression.

In a different RA model (CIA), driven by a different antigen, and a Th17 CD4 T cell response, a J-CII conjugate vaccine (CEL-2000) also reduced disease severity and serum levels of IL-17 in mice (61). This highlights the importance of determining which Th pro-inflammatory cytokines (Th17 in CIA) are driving the disease in order to utilize the appropriate LEAPS therapy to rebalance the immune system and obtain a favorable response to LEAPS therapy.

Studies were also performed on a second DerG-LEAPS vaccine that incorporated a different PG peptide representing a citrullinated PG epitope (DerG-PG275Cit; CEL-5000) (**Table 2**). Citrullinated proteins are considered to be neoantigens that, through elicitation of autoAbs and Th cells, may trigger the onset of disease in RA patients (62). The PG275Cit alternative peptide was also investigated to provide an additional epitope for individuals with a different T cell receptor (TCR) specificity. Although initial human studies are likely to be performed on CEL-4000 only, having two therapeutic LEAPS vaccines offers an opportunity to switch therapies for an individual or to combine the therapies to have broader antigenic coverage in a future immunomodulatory treatment of RA. CEL-5000 was tested alone and in combination with CEL-4000 in mice with GIA. The progression of arthritis was curtailed in mice treated with either CEL-5000 alone or in combination with CEL-4000. Interestingly, the humoral immune response to CEL-5000 alone was different from that to CEL-4000 (6).

**Table 2:** Effects of CEL-4000 peptide on ex vivo differentiated and stimulated CD4^+^ T cells from mice with GIA or from naive mice.

	Concentrations of key cytokines secreted ex vivo by rhG1-stimulated T helper (Th) cells from mice with GIA			
Th Cell Subset- Peptide	IL-4	IL-10	IFNγ	IL-17A
	pg/mL (Mean ± SEM)	pg/mL (Mean ± SEM)	pg/mL (Mean ± SEM)	pg/mL (Mean ± SEM)
**Th0 - None**	1032 ± 429	2550 ± 885	187 ± 45	38 ± 15
**Th0 - CEL-4000**	769 ± 291	2100 ± 885	429 ± 234	46 ± 8
**Th1 - None**	135 ± 44	365 ± 97	1504 ± 351	25 ± 5
**Th1 - CEL-4000**	121 ± 39	497 ± 134	1885 ± 225	29 ± 8
**Th2 - None**	926 ± 380	1797 ± 380	53 ± 28	63 ± 12
**Th2 - CEL-4000**	11865 ± 6039*	9286 ± 1376*	151 ± 74	60 ± 11
**Th17 - None**	107 ± 36	410 ± 132	69 ± 34	9145 ± 918
**Th17 - CEL-4000**	83 ± 26	479 ± 164	197 ± 71	12482 ± 25
	Concentrations of key cytokines secreted ex vivo by anti-CD3/CD28-stimulated Th cells from naïve mice			
Th Cell Subset- Peptide	IL-4	IL-10	IFNγ	IL-17A
	pg/mL (Mean ± SEM)	pg/mL (Mean ± SEM)	pg/mL (Mean ± SEM)	pg/mL (Mean ± SEM)
**Th0 - None**	73049± 16002	13345 ± 1648	3014 ± 521	73 ± 33
**Th0 - CEL-4000**	67923 ± 8422	13294 ± 1983	2888 ± 319	75 ± 29
**Th1 - None**	13837 ± 1616	2180 ± 483	25832 ± 6711	66 ± 34
**Th1 - CEL-4000**	12547 ± 1114	2529 ± 508	25912 ± 6672	68 ± 36
**Th2 - None**	145624 ± 19353	31986 ± 3695	2935 ± 597	76 ± 34
**Th2 - CEL-4000**	180698 ± 20398	30490 ± 4519	2632 ± 469	97 ± 41
**Th17 - None**	338 ± 47	2049 ± 171	3256 ± 421	11351 ± 1995
**Th17 - CEL-4000**	352 ± 73	1879 ± 50	3318 ± 484	13135 ± 2839

* Demonstration of activity.

Data adapted from Figures S3 and S4 in reference (6). CD4^+^T helper (Th) cells were prepared from the spleens of BALB/c female mice either immunized for GIA (top panel) or non-immunized (naïve) animals (bottom panel) using an immunomagnetic CD4 T cell separation kit and negative selection. Antigen presenting cells, when used (top panel), were prepared from separate aliquots of GIA spleen cells by depleting T cells via positive immunomagnetic depletion with a biotinylated anti-CD3 antibody. The undifferentiated CD4^+^ T (Th0) cells were differentiated in culture (using R&D Systems’ appropriate CellXVivo kit reagents) into Th1, Th2, or Th17 subsets, or left without reagent for undifferentiated Th0 cells (both panels). The cells were stimulated with rhG1 in the case of GIA donor cells (top panel) or with polyclonally activating anti-CD3/CD28 mAbs for Th cells from naïve donors (bottom panel). CEL-4000 peptide was absent (No peptide) or present (CEL-4000) during differentiation and for 2 days after differentiation to evaluate the effect of CEL-4000 on the undifferentiated (Th0) or differentiated (Th1, Th2, Th17) cells from mice with GIA (top panel) or cells from naïve mice (bottom panel). Cytokine production was determined by Multiplex (9-plex, 8-plex, or 6 plex) by R&D Systems’ Magpix method and results are expressed as concentrations. ^*^ Bold, italicized, underlined numbers (top panel) demonstrate the effects of CEL-4000 on the production of the anti-inflammatory/regulatory cytokines IL-4 (~5 fold greater than no peptide control) and IL-10 (~5-fold greater than control) byTh2 cells from GIA mice. Polyclonally activated cells from naïve mice (bottom panel) did not show such antigen-specific effects.

**Figure 3:**
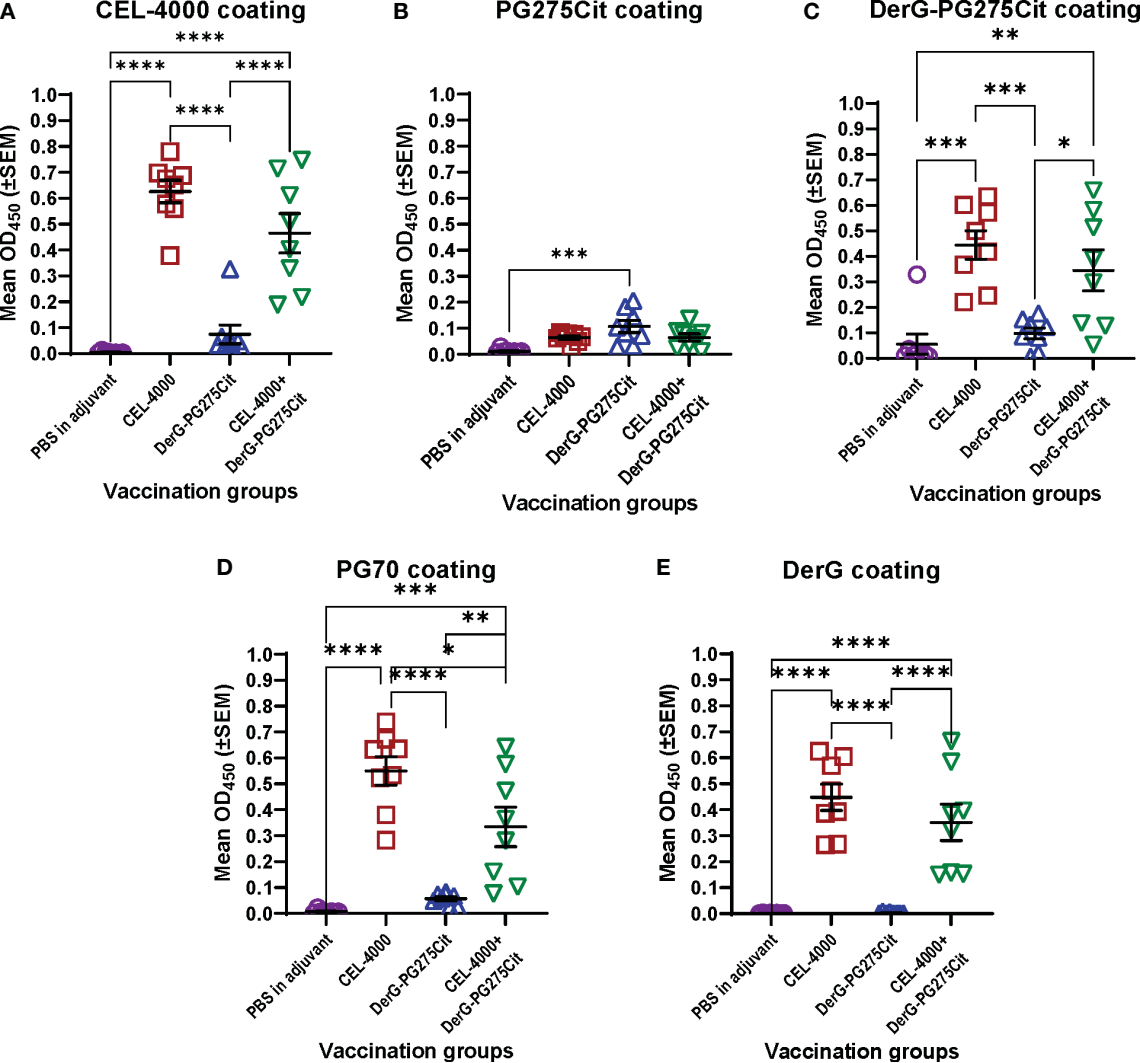
Serum antibody responses of mice with GIA after vaccination with adjuvant (control) or CEL-4000, DerG-PG275Cit (CEL-5000) or the combination of CEL-4000 and CEL-5000. Mouse serum samples (at 1:1000 dilution) were reacted with plate-immobilized CEL-4000 (DerG-PG70) **(A)**, PG275Cit **(B)**, DerG-PG275Cit **(C)**, PG70 **(D)** or DerG **(E)** peptides. IgG antibodies bound to the peptides were detected with anti-mouse IgG antibody. The optical density (OD) was determined at 450 nm using an ELISA reader. Data are expressed as the mean ± SEM (n = 8 mice/group; data analyzed using one-way ANOVA followed by Tukey`s multiple comparison test. *p<0.05; **p<0.01; ***p<0.001; ****p<0.0001). Data adapted from Figure 6 in reference (6).

As we stated in a previous PG epitope-related study (63), the PG70 epitope sequence differs, but the PG275 epitope sequence is identical, between humans and mice. Mice with either PGIA or GIA recognize PG70 as a “foreign” epitope, but poorly recognize the PG275 “self” epitope. This was also reflected in the serum Ab titers, as the GIA mice did not produce much antibody against either the native or the citrullinated form of the PG275 epitope but made Abs to the PG70 epitope (6) (**Figure 3**). Even though mice with GIA produced Abs only in response to vaccination with CEL-4000 (or with the combination of CEL-4000 and CEL-5000), both therapies were effective because the immune modulation was through T cells and the anti-CEL-4000 Abs (shown in **Figure 3**) probably did not neutralize the CEL-4000 vaccine. In a previous PG epitope-related *in vitro* study on RA patient cell and serum samples, most of the patient samples showed reactivity (T cell and Ab responses) to both the PG70 and PG275 epitopes, especially to the citrullinated version of PG275 (63), suggesting that treatment with CEL-4000 and CEL-5000 vaccines (alone or in combination) would be effective in a certain proportion of patients with RA.

The classical approach to demonstrating an immune response to most vaccines [see the guidance document including the references by US FDA (64)] is to examine anti-vaccine Ab titers. CEL4000 was tested for safety and anti-vaccine responses in healthy non-human primates (NHP, cynomolgus monkeys) by Altasciences, Co, Inc, (Laval, Quebec, Canada, and Everette, WA, USA), an independent research agency. Altasciences is a Good Laboratory Practice (GLP) practicing Contract Research Organization (CRO). They reported detection of anti-CEL-4000 Abs, but no T cell immune responses to vaccination (data available from Altasciences and on file), as would be expected for a nondiseased animal. This demonstrates that, for the LEAPS vaccines, anti-peptide Abs may actually be inappropriate and unrevealing as indicators of therapeutic efficacy. Better surrogate markers for both the CEL-4000 or a combined CEL-4000/CEL-5000 vaccine treatment efficacy would be changes in cytokine profiles indicative of restored immune regulation as well as reduction of disease symptoms.

As an example of how surrogate markers can be used, the immunomodulatory effects of CEL-4000 or the combination of CEL-4000 and CEL-5000 vaccines were demonstrated on established effector/memory T (Teff/Tm) cell responses. T cells of mice with GIA were treated *in vitro* with the immunizing antigen (rhG1) to expand antigen-specific Teff and Tm cell populations in culture, then treated to promote their differentiation into Th1, Th2 or Th17 cells or left untreated (for Th0 undifferentiated cells) prior to *in vitro* treatment with the CEL-4000 peptide (**Table 2**). As expected from earlier studies on the DerG (or G) ICBL (65–68), CEL-4000 had an effect on Th2 and Treg cells *in vivo* (6, 7), which was demonstratable herein *in vitro* on antigen-experienced differentiated Th2 cells by the increased production of IL-10 and IL-4 (a regulatory and an anti-inflammatory cytokine, respectively), but no similar effect was noted for the Th0, Th1, or Th17 cells obtained from mice with GIA (**Table 2**, top panel). In addition, as shown in **Table 2** (bottom panel), CEL-4000 treatment did not affect similarly differentiated T cells from naïve mice (of the same sex and strain) when these cells were treated with the polyclonal activator anti-CD3/anti-CD28 mAbs *in vitro* (6). Antigen specificity of the PG70 peptide and the importance of the DerG ICBL in CEL-4000 was demonstrated by the lack of response to a PG70 conjugate incorporating a different ICBL (J- PG70) and to a DerG peptide attached to an irrelevant influenza virus epitope, respectively (6). By acting on Th2 (and possibly Treg) cells in these *in vitro* experiments, CEL-4000 appeared to provide an enhanced and antigen-specific regulatory mechanism by modulating the profile and function of Tm/Treg cells obtained from mice with GIA.

## 4 Recommendation for adaptive design clinical studies of CEL-4000 vaccine

Designing or planning, and possibly conducting, phase I studies for vaccine-based immunomodulation (i.e., immunotherapy) for ongoing RA requires a different approach than a preventive vaccine. For a preventive vaccine administered to an “immunonaive” individual for protection against infection, once safety has been determined, efficacy can be indicated by the presence and titers of neutralizing anti-microbial Abs, which can be readily assayed. T cell responses and associated cytokines are secondary findings even if they are important for protection. For a therapeutic vaccine administered to a patient with RA, the relevant findings, in addition to lessening of the disease symptoms, are the changes in T cell responses and/or specific cytokine production, the values of which will be presumably different in each patient.

Evaluation of the efficacy of therapies for RA (as for cancer, for example) is only relevant in disease-bearing animals or individuals. In all cases, efficacy of the treatment is indicated by curtailment of the progression or by accelerated resolution of the disease. Therefore, relevant human testing of the CEL-4000 vaccine must be performed on individuals with RA. Ideally, testing would be performed on patients with early-stage RA by demonstrating reduced disease progression as well as changes in immunological parameters as surrogate indicators of a successful provision of therapy. For such a vaccine, adaptive design studies, commonly used for testing cancer therapies on diseased patients (even for phase I First in Human/First in Man (FIH/FIM) studies), would be most appropriate, as they are considered more ethical and efficient than standard phase I trials with healthy volunteers.

**Figure 4:**
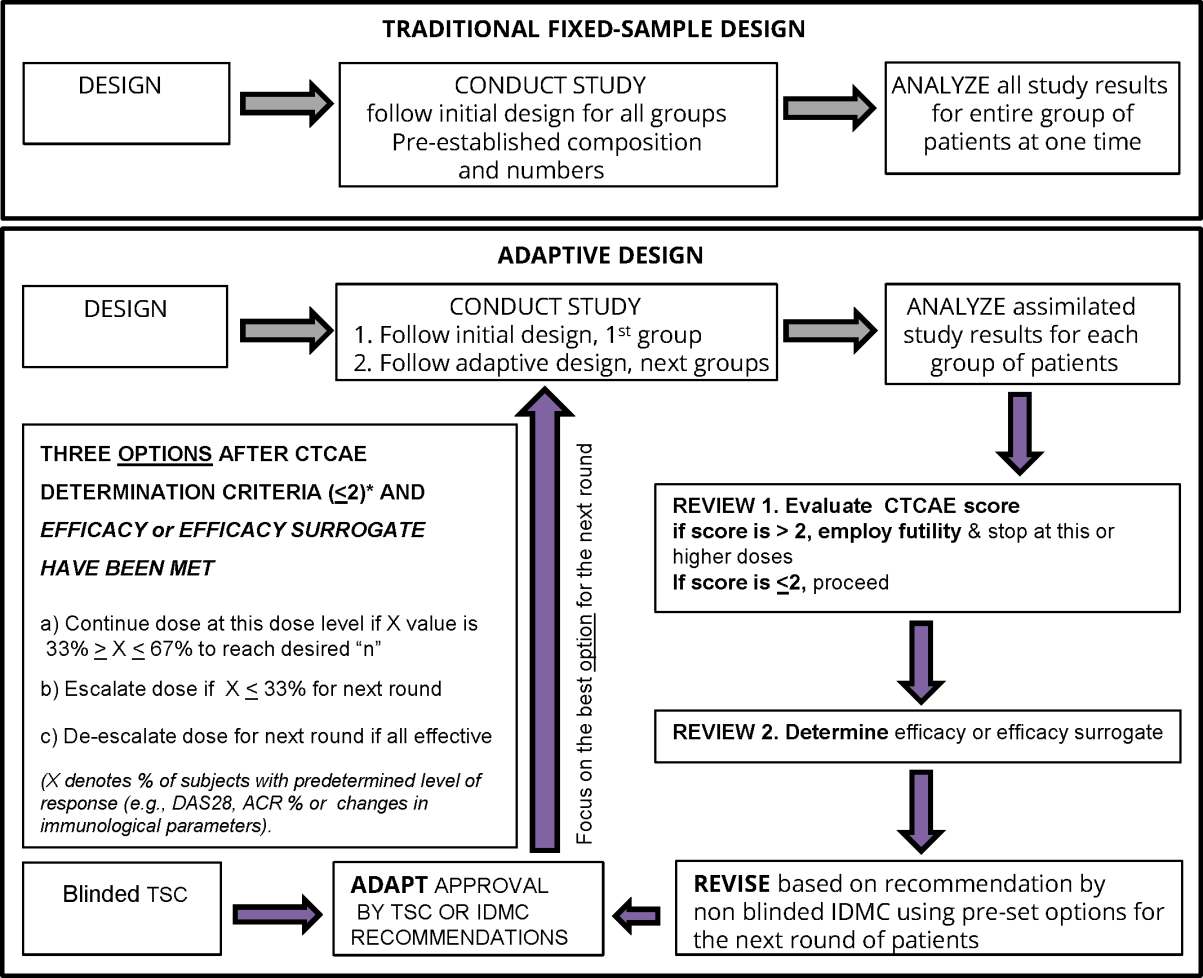
Bayesian optimal interval (BOIN) adaptive design study proposed for a clinical trial of therapeutic vaccines such as CEL-4000 for RA. The figure is adapted from **Figure 1** in Pallman et al. (69). The decision steps for a conventional (traditional fixed-sample) and a BOIN study are compared. For the BOIN adaptive study, if the 1st step passes (for safety or toxicity) with CTCAE <2 then continuation proceeds to the 2nd step for determination of efficacy or a surrogate of efficacy. The criteria for the 4 options for continuing to the next round are listed in the largest box in the left side of the figure. The options are based on tests that determine the percent of responders (X) as indicated by pre-determined rules for evaluating disease activity (DAS28*or ACR* response %), toxicity, or treatment efficacy either by response rate with a pre-determined X value or favorable surrogate efficacy parameters (e.g. biomarker indicative of positive result such as decreased inflammatory (TNFα, IFNγ, IL-17) or increased anti-inflammatory (IL-4, IL-10, TGFβ) levels and/or favorable ratios and/or specific numbers of appropriate cell types (e.g., Th1, Th2, Th17, Treg) or other select biomarkers). Abbreviations: ACR, American College of Rheumatology; CTCAE, Common Criteria Terminology for Adverse Events (77); DAS28, Disease Activity Score for 28 joints; IDMC, Independent Data Monitoring Committee; TSC, Trial Safety Committee.

In designing Phase 1 studies for CEL-4000, we reviewed several studies and websites for guidance, especially those that were carried out for RA, and those employing adaptive design efficacy-based studies (69–76). As shown in **Figure 4** (as modified from Pallman et al. (69)), an adaptive design study proceeds with concurrent analysis of safety and efficacy based on agreed-upon rules to allow progression of the study (determined prior to trial initiation) and implemented by an independent drug safety monitoring board. For example, with a low number of subjects treated with a single dose of vaccine, specific steps (Design, Conduct, Analyze, Review and Adapt) are established, and data is analyzed and used to determine the optimal treatment regime for the next round or cycle. Adaptive design studies allow a change in dose during the course of a trial, based on safety or efficacy parameters. If a dose is judged to be unsafe, based on a > 2 serious adverse event (SAE) score, then it is futile to continue, and a futility designation is made to discontinue treatments at the given or higher doses. However, if a dose is judged as safe i.e., having a ≤ 2 SAE score, then the study can proceed to examine the parameters of efficacy or surrogates of efficacy in the same subjects. Notably, futility consideration can also occur with acceptable safety profiles if immune response or efficacy criteria are not met, but this scenario would allow an increase in dosing or frequency of administration within safety limits, instead of study termination.

The adaptive design is best demonstrated and formalized in the Bayesian optimal interval (BOIN) studies (78–82). The advantage of the adaptive or Bayesian design approach is that it allows examination of both the safety/toxicity and efficacy parameters (lessening of the symptoms or surrogates of efficacy, as criteria) all at the same time and with pre-established and agreed-upon rules for discontinuation or redirection at specified points as the study progresses. The BOIN approach is recognized by the US FDA and European Medicines Evaluation Agency (EMEA) authorities as appropriate for certain FIH/FIM studies and has been used in oncology clinical trials for new peptide therapies including peptide vaccines (78–83). Bayesian design is based on a method of statistical inference in which Bayes’ theorem is used to update the probability (p value) for a hypothesis being tested as more evidence or information becomes available, e.g., after the start of the treatment of the subjects enrolled in the study. The FDA is encouraging the use of the adaptive design, such as the BOIN approach, because it is more ethical (providing therapy and demonstrating safety and non-toxicity in diseased instead of healthy individuals) and often requires fewer subjects by not continuing if prevalent futility elements are present.

For CEL-4000, the emphasis of the first trial on RA patients would be on the safety, and lack of toxicity, including immunotoxicity (e.g., an unexpected cytokine storm). If these criteria are met, the study would then proceed to the second step of efficacy, as indicated by reduction of symptoms or a surrogate indication of therapeutic success, such as (non-neutralizing) Ab production to CEL-4000 and changes in the production of cytokines (or ratios) or the types of T cells indicative of beneficial immunomodulation. Although the adjuvant (manufactured by Seppic and called ISA51vg or Montanide) used in our LEAPS vaccine formulations has proven safe in numerous human trials for cancer vaccines (including the ones using peptides similar to CEL-4000) for many years (see recent review by Melssen et al. (84)), it may also be necessary to evaluate the adjuvant’s safety separately.

It is proposed that subject selection will follow the most recent recommendations of the EULAR and the ACR for the management or RA using synthetic and biological DMARDs (13, 14) as approved by the study sponsor’s independent data monitoring committee (IDMC). Further, appropriate inclusion and exclusion criteria will be applied. The subject selection/inclusion criteria and the study design will have to be approved and performed at an appropriate clinical site.

The adaptive or Bayesian design with the BOIN approach seems to be the best for testing an immunomodulatory therapy, such as the CEL-4000 vaccine, in RA patients for several reasons (**Figure 4**). It would allow examination of numerous parameters at the same time, e.g., safety and toxicity, while also setting milestones for efficacy (beneficial changes in symptoms) or surrogates of efficacy (e.g., reduction of pro-inflammatory cytokines, and/or increase in anti-inflammatory/regulatory cytokines and Tregs). If a dose is judged as not safe, a futility designation would be made, and the study at that dose would be discontinued and possibly a lower dose would be tested. However, if a dose is judged as safe, then the study would proceed to examine efficacy or a surrogate of efficacy in the same subjects, and if appropriate, to consider an escalation or de-escalation of the dose in the next round of the study (77). The adaptive study design can also be applied to immunological parameters for determination of the best surrogate biomarkers indicative of treatment efficacy. These decisions can be made on the same day when the safety review is performed by the IDMC. The BOIN approach would be more economical, efficient, and compassionate for the participants than the traditional, fixed-sample study design (**Figure 4**).

Refinement of the CEL-4000 vaccine study would include various considerations, e.g., the criteria for patient inclusion or exclusion for the study, specific details regarding the futility designations, and potential comparisons with established RA treatments. Patient inclusion may potentially include screening for the presence of T cell cytokines that are promoting inflammation or other ex vivo parameters indicative of T cell and/or Ab reactivity to the vaccine if resources of such tests are available. **Table 3** shows some of the risk factors and established laboratory findings related to RA for which the potential study population could be screened. In addition to age, gender, and smoking status, the patient’s HLA alleles, and RA-specific laboratory parameters (85–87) can be reviewed, keeping in mind that RA is a heterogeneous disease. Unfortunately, the field of RA-related biomarker tests has not advanced as fast as desired in order to allow identification of the best biomarkers to focus on at this stage of product development. For further insights, the reader is referred to the description of a RA activity biomarker test (MBNA Vectra®, now provided by LabCorp) (85–87) and a RA genetics review (88).

**Table 3:** Risk factors and laboratory parameters to consider in a BOIN study population for testing CEL-4000 in RA patients.

• **Age**, Incidence: young < elderly, increases with age progression
• **Gender**, Incidence: female > male (approximately 3-fold difference)
• **Ethnicity**, Incidence: Native Americans > Old world immigrants; Western, Northern > Eastern, Southern European regions of origin
• **Family history**, Incidence: strong association with autoimmune conditions in family
• **Smoking status**, Incidence: strong association with smoking
• **Periodontal disease**, Incidence: strong association with the presence of periodontal disease
• **Rural or urban habitat**, Incidence: urban > rural
• **Genetic and/or epigenetic risk factors**, Incidence: increased in the presence of risk factors (also see RA-specific SNPs below)
• **Shared Epitope (SE) and subgroups of SE**, Incidence: S2 > S3P >S3D >S1
• **HLA-DRB alleles**, Incidence: increased for DRB1*01, DRB1*04, DRB1*10, sometimes for DRB13 and DRB15
• **ACPA (anti-citrullinated protein antibody) positivity**, Incidence: increased above 25 U/ml in serum, or above the minimum level defined in each cyclic citrullinated peptide (CCP) antibody assay kit
• **RF (rheumatoid factor) positivity**, Incidence: increased in the presence of RF in serum
• **Cytokines**, Incidence: increased if pro-inflammatory (IL-1, -6, -17, IFNγ and TNFα) cytokines outbalance the anti-inflammatory (IL-4, IL-10, TGFβ) cytokine concentrations (see below for available tests)
• **ESR (erythrocyte sedimentation rate), CRP (C-reactive protein), RF, ACPA and additional RA biomarker tests by Vectra/LabCorp or comparable supplier)**, Incidence: increased if elevated levels of ESR, CRP, RF, and ACPA are detected, in addition to 12 biomarkers by Vectra DA or similar multicomponent biomarker tests
• **SNPs (single nucleotide polymorphisms) in genes or chromosomal regions**, Incidence: increased for certain genes or chromosomal loci: *PTPN22* > *TRAF1-C5* > *PADI1 or 4* > other *PADI*. Additional SNPs in *IRAK2* (IL1R) rs708035, rs3844283, and *TAGAP* rs2451258
• **PG70** (proteoglycan epitope 70) or other epitope peptide (e.g., PG70Cit, PG275, PG275Cit) reactivity to these peptides can be detected in most RA patients using published antibody- or cell-based reactivity assays

Numerous genetic, epigenetic, immunological, and other risk factors might influence the phenotype of RA. This could influence the outcomes of any preventive or therapeutic interventions in RA. Healthy volunteers (with no RA risk factors and no subclinical signs of inflammation) could be excluded from a therapeutic vaccine study, since they are not likely to have many antigen-specific T memory cells that would demonstrate, or benefit from, the effects of CEL-4000 vaccine on the immune system, particularly the involvement of Th2, Treg cells and various anti-inflammatory cytokines. See the article text and references therein for more details.

## 5 Conclusions

The current therapeutic arsenal for the treatment of RA mainly consists of immunosuppressive or ablative drugs, which carry the risk of facilitating recurrent or primary infectious diseases or cancer. A therapeutic vaccine that can rebalance inflammatory disease-promoting T cell immune responses in an antigen-specific manner is highly desirable. Evaluation of a treatment for an autoimmune inflammatory disease such as RA can only be performed in individuals who have the disease. Non-diseased but at-risk individuals (e.g., siblings of RA patients showing the presence of risk factors or laboratory-determined parameters indicative of subclinical RA) may also be appropriate study subjects. However, patient selection is not straightforward, as RA phenotypes show great heterogeneity and are under the influence of numerous environmental, genetic, and epigenetic factors. Treatment efficacy in an adaptive study would ultimately be demonstrated by delayed or reduced disease progression, but also by favorable changes in surrogate immune responses indicative of an antigen-specific decrease in pro-inflammatory and/or an increase in anti-inflammatory cytokines and corresponding increase in the number or function of Treg cells. Immunomodulation by CEL-4000 will likely occur in individuals already possessing disease promoting and antigen-specific Teff/Tm cells requiring testing in these individuals. Ultimately, an antigen-specific immunomodulating vaccine, such as CEL-4000, should deliver therapy for RA without weakening important immune defense mechanisms against microbial infections or cancer.

## Author contributions

DZ: Conceptualization, Funding acquisition, Investigation, Project administration, Supervision, Writing – original draft, Writing – review & editing. ZS: Formal analysis, Visualization, Writing – review & editing. AM: Conceptualization, Data curation, Formal analysis, Investigation, Methodology, Validation, Visualization, Writing – original draft, Writing – review & editing. KSR: Conceptualization, Data curation, Formal analysis, Investigation, Writing – original draft, Writing – review & editing. RC: Data curation, Formal analysis, Investigation, Software, Writing – review & editing. KM: Conceptualization, Data curation, Formal analysis, Funding acquisition, Project administration, Visualization, Resources, Writing – original draft, Writing – review & editing.
